# Beurteilung des Stellenwertes der neuropädiatrischen Diagnostik im Rahmen der initialen Autismusabklärung

**DOI:** 10.1007/s10354-023-01012-w

**Published:** 2023-05-03

**Authors:** Sarah Ruffing, Christine Ullrich, Marina Flotats-Bastardas, Martin Poryo, Sascha Meyer

**Affiliations:** 1 Pädiatrische Kardiologie, Universität des Saarlandes, Kirrberger Str., Geb. 9, 66421 Homburg/Saar, Deutschland; 2 Klinik für Allgemeine Pädiatrie und Neonatologie, Universität des Saarlandes, Homburg/Saar, Deutschland

**Keywords:** Autismus-Spektrum-Störung, Tiefgreifende Entwicklungsstörungen, Neuropädiatrische Untersuchung, Neuropädiatrische Diagnostik, Standard operating procedure, Autism spectrum disorder, Pervasive developmental disorders, Neuropediatric assessment, Neuropediatric diagnostics, Standard operating procedure

## Abstract

**Hintergrund:**

Die Diagnostik bei Autismus-Spektrum-Störungen ist aufgrund fehlender biologischer Marker und zahlreicher Komorbiditäten anspruchsvoll. Ziel dieser Arbeit war es, den Stellenwert der neuropädiatrischen Diagnostik zu beurteilen und eine interne Leitlinie zu erstellen.

**Methodik:**

Eingeschlossen wurden alle Patienten, die sich zwischen 04/2014 und 12/2017 in der neuropädiatrischen Ambulanz am Universitätsklinikum des Saarlandes mit der Diagnose „tiefgreifende Entwicklungsstörungen“ (ICD-Code F84) vorgestellt haben.

**Ergebnisse:**

Die Studie umfasste 82 Patienten (männlich 78 %, weiblich 22 %; Durchschnittsalter 5,9 ± 2,9 Jahre, Spanne 2 bis 16 Jahre). Häufigste Untersuchung war die Elektroenzephalographie (EEG) (74/82; 90,2 %); diese war bei 33,8 % (25/74) auffällig. Anhand der Anamnese und/oder des EEGs wurde bei 16/82 (19,5 %) Kindern die Diagnose „Epilepsie“ gestellt. Eine kranielle Magnetresonanztomographie (cMRT) erhielten 49/82 (59,8 %) der Patienten; 22/49 (44,9 %) zeigten mindestens einen auffälligen Befund; bei 14/22 (63,6 %) ließen sich eindeutige Pathologien feststellen. Eine Stoffwechseldiagnostik wurde bei 44/82 (53,7 %) Kindern veranlasst; bei 5/44 (11,4 %) resultierte daraus eine Diagnose oder der Verdacht auf eine Stoffwechselerkrankung. Das Ergebnis einer genetischen Diagnostik lag bei 29/82 (35,4 %) Kindern vor mit Auffälligkeiten in 41,4 % (12/29). Eine motorische Entwicklungsverzögerung war häufiger mit Komorbiditäten, EEG-Auffälligkeiten, Epilepsie und Auffälligkeiten in der Stoffwechsel- sowie genetischen Diagnostik assoziiert.

**Schlussfolgerung:**

Die neuropädiatrische Mitbeurteilung bei Verdacht auf Autismus sollte bei jedem Kind eine detaillierte Anamnese, eine neurologische Untersuchung sowie ein EEG beinhalten. Die Durchführung einer cMRT, einer Stoffwechsel- sowie einer genetischen Diagnostik wird nur bei klinischer Indikation empfohlen.

## Abstract (en)

**Background:**

The diagnostics of autism spectrum disorder is complex due to missing biological markers and numerous comorbidities. The aim was to assess the role of neuropediatric diagnostics and to develop a standard operating procedure for a targeted assessment.

**Method:**

All patients presenting to the neuropediatric outpatient clinic at Saarland University Hospital between April 2014 and December 2017 with ICD code F84 pervasive developmental disorders were included.

**Results:**

A total of 82 patients were included (male 78%, female 22%; mean age 5.9 ± 2.9 years, range 2–16 years). The most frequent examination was electroencephalography (EEG) (74/82; 90.2%) with pathological findings in 33.8% (25/74). Based on the history and/or EEG epilepsy was diagnosed in 19.5% (16/82). Magnetic resonance imaging (MRI) was performed in 49/82 (59.8%) patients, 22/49 (44.9%) showed at least 1 cerebral abnormality and definite pathologies could be detected in 63.6% (14/22). A metabolic diagnostic work-up was performed in 44/82 (53.7%) cases and in 5/44 (11.4%) it resulted in a diagnosis or suspicion of a metabolic disease. Genetic testing results were available in 29/82 (35.4%) children and 12/29 (41.4%) showed abnormal results. Delay in motor development was more frequently associated with comorbidities, EEG abnormalities, epilepsy and abnormalities in metabolic and genetic testing.

**Conclusion:**

Neuropediatric examination in cases of suspected autism should include a detailed history, a thorough neurological examination and an EEG. An MRI, comprehensive metabolic and genetic testing are only recommended if clinically indicated.

Der Begriff „Autismus“ bezeichnete ursprünglich das Symptom der starken Zurückgezogenheit im Rahmen einer Schizophrenie [7]. Aus dieser Beschreibung hat sich das Krankheitsbild der heutigen Autismus-Spektrum-Störung (ASS) (ICD-10 Code F84.-) entwickelt [17]. Die Kernsymptomatik mit Beginn meist im Kleinkindes‑/Kindesalter besteht aus Störungen im Bereich der sozialen Interaktion und Kommunikation und eingeschränkten, repetitiven Verhaltensmustern und Interessen. Veränderungen werden nur schwer toleriert, und es besteht ein Bedürfnis nach bekannten Ritualen. Die ASS stellt ein sehr heterogenes Krankheitsbild dar, welches von milden Symptomen bis hin zu einer schwerwiegenden Einschränkung mit der Notwendigkeit einer lebenslangen Unterstützung reicht.

Die weltweite Prävalenz der ASS wird auf ca. 0,62–1 % geschätzt [9] mit einer deutlichen Geschlechtswendigkeit hin zum männlichen Geschlecht (männlich zu weiblich: 2–5:1) [15, 17, 22]. Weibliche Betroffene bleiben aber auch oft unerkannt oder werden erst spät diagnostiziert, da sie häufig über bessere Anpassungsfähigkeiten verfügen und vermutlich auch hormonelle Ursachen wie der Einfluss von fetalem Testosteron von Bedeutung sind [18, 38].

Die Ursachen der ASS sind noch immer nicht vollständig geklärt. Am ehesten handelt es sich um eine multifaktorielle, stark genetisch determinierte Entwicklungsstörung des zentralen Nervensystems (ZNS). In ca. 10 % der Fälle liegt ein syndromaler Autismus vor z. B. im Rahmen einer tuberösen Sklerose, eines Fragile-X-Syndroms, einer Neurofibromatose oder eines Rett-Syndroms [23, 25, 30, 32]. Daneben spielen aber auch prä-, peri- und postnatale Umwelteinflüsse eine Rolle [40]. So erhöhen beispielsweise eine Frühgeburtlichkeit und ein niedriges Geburtsgewicht das Risiko für neurologische Entwicklungsstörungen und somit auch für autistische Störungen [31].

Die Diagnostik der ASS wird dadurch erschwert, dass es bisher keinen spezifischen Test oder biologischen Marker für die Diagnosestellung gibt. Daher ist ein multidisziplinäres Team erforderlich, um eine differenzierte Diagnostik durchzuführen und die Diagnose einer ASS stellen zu können [8]. Bei klinischem Verdacht werden zunächst Fragebögen als Screeninginstrumente eingesetzt („Modified Checklist for Autism in Toddlers“ für Kinder ab 18 Monaten, „Fragebogen zur sozialen Kommunikation“ [FSK] für Kinder ab 4 Jahren) [2, 17, 31]. Bei einem positiven Screeningergebnis oder starkem klinischem Verdacht sollte dann eine Diagnostik bezüglich des Vorliegens einer ASS erfolgen [7]. Goldstandard in der Diagnostik der ASS sind das „Autism Diagnostic Interview-Revised“ (ADI-R) und das „Autism Diagnostic Observation Schedule“ (ADOS) [2, 7, 19].

Erschwert wird die Diagnostik dadurch, dass viele komorbide Störungen und Symptome, wie z. B. Aufmerksamkeitsprobleme oder Intelligenzminderung, auch als Differenzialdiagnose der ASS in Betracht kommen [35]. Neben der spezifischen Diagnostik der ASS sollten deshalb zusätzliche Untersuchungen durchgeführt werden, um mögliche Komorbiditäten und Differenzialdiagnosen zu detektieren sowie insbesondere psychiatrische Störungen auszuschließen. Hierzu gehört u. a. eine differenzierte internistisch-neurologische Untersuchung: Bei klinischen Auffälligkeiten wie einer Mikro- oder Makrozephalie, Krampfanfällen oder neurologischen Symptomen sind weitere Untersuchungen wie eine Elektroenzephalographie (EEG), eine kranielle Magnetresonanztomographie (cMRT), Stoffwechsel- und/oder genetische Untersuchungen indiziert [13]. Darüber hinaus müssen somatische Ursachen als mögliche Auslöser einer sprachlichen und sozialen Beeinträchtigung ausgeschlossen werden, weshalb auch eine augenärztliche sowie HNO-ärztliche Abklärung notwendig sind [13, 26].

Patienten mit ASS haben ein erhöhtes Risiko für eine Epilepsie (Altersgipfel < 5. und > 10. Lebensjahr) [36]. Die Prävalenz liegt hier je nach Untersuchung bei 8–30 %, wobei aber rund ein Drittel der Patienten mit epilepsietypischen Auffälligkeiten im EEG keine klinischen Krampfanfälle aufweisen [16, 20]. Faktoren wie eine Intelligenzminderung, neurogenetische Erkrankungen, das weibliche Geschlecht, Entwicklungsrückschritte und das Alter werden als Einflussfaktoren für das Auftreten einer Epilepsie im Rahmen einer ASS diskutiert [16, 20].

Pathologische Befunde in der cMRT können bei 6,5–26,2 % der Patienten mit ASS erhoben werden. Hierbei steigt die Wahrscheinlichkeit für das Auftreten pathologischer Befunde, wenn die cMRT aufgrund von Auffälligkeiten in der neurologischen Untersuchung, Kopfschmerzen oder Krampfanfällen veranlasst wird [6]. Bei ca. 15–20 % der Kinder mit ASS kann zudem eine Makrozephalie nachgewiesen werden [28]. Levy et al. fanden bei diesen Patienten gehäuft vergrößerte Temporal- und Frontallappen sowie eine vergrößerte Amygdala – Regionen, die für die Entwicklung sozialer, kommunikativer und motorischer Fähigkeiten von Bedeutung sind [21].

Bisherige Untersuchungen zeigen, dass angeborene Stoffwechselerkrankungen bei Personen mit ASS selten sind [24, 33]. Da bei einigen Stoffwechselerkrankungen durch diätetische oder medikamentöse Maßnahmen die Symptome der ASS aber positiv beeinflusst werden können, empfehlen einige Autoren dennoch im Rahmen der Abklärung einer ASS den Ausschluss einer Stoffwechselerkrankung [12], auch wenn die aktuell gültige AWMF-Leitlinie keine routinemäßigen Laboruntersuchungen dieser Art empfiehlt [7]. Ebenfalls kontrovers diskutiert wird die Durchführung genetischer Tests. Bei 6–40 % der Patienten mit ASS lässt sich eine genetische Ursache identifizieren [10, 32]. Die Durchführung einer genetischen Untersuchung bietet die Möglichkeit, die Ätiologie zu klären als auch Informationen bezüglich der Prognose, des Wiederholungsrisikos sowie möglicher Therapien zu gewinnen.

Ziel der vorliegenden Arbeit ist es, den neuropädiatrischen Diagnoseprozess und das Patientenkollektiv mit ASS aus neuropädiatrischer und epidemiologischer Sicht darzustellen. Aktuell wird an unserer Klinik eine sehr breite Abklärung dieser Patienten durchgeführt und weiterführende Diagnostik großzügig angewandt. Inwiefern hierbei pathologische und behandlungsbedürftige Befunde hervorgehen, soll dargelegt werden. Zudem soll für das diagnostische Vorgehen ein standardisiertes Vorgehen („standard operating procedure“ [SOP]) etabliert werden, um eine zielgerichtete Abklärung, auch bezüglich des Vorliegens relevanter Komorbiditäten, zu ermöglichen.

## Material und Methodik

Es handelt sich um eine retrospektive Studie am Universitätsklinikum des Saarlandes, Klinik für Allgemeine Pädiatrie und Neonatologie, die am 17.05.2019 durch die Ethik-Kommission der Ärztekammer des Saarlandes (Kenn-Nr. 105/19) bewilligt wurde. Eingeschlossen wurden alle Patienten unter 18 Jahren mit der Diagnose einer ASS (ICD-10 Code F84.-), die sich im Zeitraum 01.04.2014 bis 31.12.2017 in der neuropädiatrischen Ambulanz vorgestellt hatten. Ausgeschlossen wurden diejenigen Patienten, bei denen keine Unterlagen verfügbar waren oder der Vorstellungsgrund nicht die Abklärung einer ASS war.

Zur Datenerhebung erfolgte die Durchsicht der physischen als auch der digitalen Patientenakten im Klinikinformationssystem (SAP, Walldorf, Deutschland) des UKS. Auf die Daten der Patienten, die nur konsiliarisch im Auftrag der Klinik für Kinder- und Jugendpsychiatrie (KJP) am UKS in der neuropädiatrischen Ambulanz gesehen wurden, konnte aufgrund fehlender Zustimmung nicht zugegriffen werden. Nicht erhobene oder nicht einsehbare Daten wurden in der Auswertung als fehlend gewertet.

Erfasst wurden Alter, Geschlecht, Datum der Erstvorstellung, Auffälligkeiten in der Schwangerschaft wie psychische Belastung, Rauchen oder Frühgeburtlichkeit und die Familienanamnese. Die Familienanamnese wurde als positiv gewertet bei Vorliegen von genetischen Erkrankungen, Epilepsie, mentaler Retardierung, Entwicklungsverzögerung/Lernschwäche sowie ASS.

Der Entwicklungsstand des Kindes wurde anhand der motorischen und sprachlichen Entwicklung sowie der Intelligenz beurteilt. Darüber hinaus wurden das Erreichen von Entwicklungsmeilensteinen und die Teilnahme des Kindes an Frühfördermaßnahmen wie Logopädie oder Ergotherapie dokumentiert.

Vorhandene Komorbiditäten wurden in somatische und psychische Erkrankungen eingeteilt. Als somatische Begleiterkrankungen wurden berücksichtigt: Epilepsie, Fieberkrampfanfall, Stoffwechselstörung, Fehlbildung, Kopfschmerzen, Herzfehler, Fußfehlhaltung, syndromale Erkrankung und Ernährungsintoleranz. Unter psychische Begleiterkrankungen fielen: Schlaf‑, Ausscheidungs‑, Essstörung, Aggressivität, geringe Frustrationstoleranz, Hyperaktivitäts- und Aufmerksamkeitsstörung sowie emotionale Störung des Kindesalters.

Die Autismusform wurde anhand der ICD-10 F84 (tiefgreifende Entwicklungsstörung) in 4 Untergruppen klassifiziert: frühkindlicher Autismus (F84.0), atypischer Autismus (F84.1) und Asperger-Syndrom (F84.5) sowie nicht klassifiziert.

Die statistische Auswertung der erhobenen Daten erfolgte mittels des Statistikprogramms IBM SPSS Statistics 25.0 für Windows (IBM, Ehningen, Deutschland). Für nominale Variablen erfolgte die Berechnung der absoluten und relativen Häufigkeiten, für kontinuierliche Variablen Mittelwert, Median und Streuung. Zudem wurden die Ergebnisse zweier Gruppen, motorische Entwicklungsverzögerung vs. normale Entwicklung, verglichen. Zum Vergleich kategorialer Zielgrößen wurde der Chi^2^-Test durchgeführt. Das Signifikanzniveau wurde mit *p* < 0,05 festgelegt.

## Ergebnisse

Vom 01.04.2014 bis 31.12.2017 wurden insgesamt 108 Patienten mit dem ICD-10 Code F84 in unserer neuropädiatrischen Ambulanz vorgestellt. Hiervon wurden 26/108 (24,1 %) von der Datenauswertung ausgeschlossen. Gründe hierfür waren: keine verfügbaren Unterlagen, Vorstellungsgrund anderer als eine Autismusabklärung sowie Ausschluss einer ASS.

Somit wurden insgesamt 82 Kinder und Jugendliche in die weitere Datenauswertung eingeschlossen (männlich 78 %, weiblich 22 %). Das Durchschnittsalter der Patienten betrug 5,9 ± 2,9 Jahre (Spannweite 2 Jahre bis 16 Jahre). Am häufigsten wurde die Diagnose einer nicht näher bezeichneten ASS gestellt (40/82; 48,8 %). Bei 30/82 (36,6 %) Kindern ließ sich eine auffällige Familienanamnese feststellen, und Auffälligkeiten in der Schwangerschaft zeigten sich bei 25,6 % (21/82) der Patienten. Bei 85,4 % (70/82) der Kinder zeigte sich eine sprachliche Entwicklungsverzögerung.

Die internistische Untersuchung ergab bei 30/78 (38,5 %) Kindern pathologische Befunde, am häufigsten Dysmorphiezeichen (13/30; 43,3 %), und bei 72/78 (92,3 %) Kindern zeigten sich Auffälligkeiten in der neuropädiatrischen Untersuchung, die insbesondere die Sprache betrafen (67/78; 85,9 %). Auffälligkeiten in der augenärztlichen Untersuchung fanden sich bei 53,1 % (17/32) der Patienten; am häufigsten (13/17; 76,5 %) waren die Kinder von einer Amblyopie betroffen.

Ein Hörtest wurde bei 43/82 (52,4 %) Kindern dokumentiert; 2/43 (4,6 %) Kinder zeigten hier auffällige Ergebnisse im Rahmen einer Schwerhörigkeit. Bei diesen Kindern lag gleichzeitig auch eine Sprachentwicklungsverzögerung vor.

Im EEG fand sich bei 25/74 (33,8 %) Kindern ein auffälliger Befund, wobei sich mehrheitlich (20/25; 80,0 %) fokale epilepsietypische Potenziale (ETP) zeigten. Insgesamt wurden 16/82 (19,5 %) Kinder mit einer Epilepsie erfasst, wobei bei 14/16 (87,5 %) das EEG eine Auffälligkeit zeigte. Bei 8/16 (9,8 %) Patienten wurde im Rahmen der Autismusabklärung eine Epilepsie erstmals diagnostiziert; bei den restlichen 8 Kindern war die Epilepsie bereits bekannt.

In der kranialen Magnetresonanztomographie (cMRT) (49/82; 59,8 %) fanden sich 30 auffällige Befunde verteilt auf 22/49 (44,9 %) Kinder (maximal 3 Auffälligkeiten pro Kind). Bei Vorliegen einer Makro- oder Mikrozephalie war das cMRT in 55,5 % (45/82) auffällig, bei Vorliegen einer Epilepsie in 53,8 % (44/82) und bei pathologischem EEG in 50,0 % (41/82).

Die Stoffwechseldiagnostik zeigte bei 13/44 (29,5 %) Patienten mindestens ein auffälliges Ergebnis. Bei 5/44 (11,4 %) Kindern wurde eine Diagnose oder der Verdacht auf eine Stoffwechselerkrankung gestellt: Phenylketonurie (*n* = 2), Leigh-Syndrom (*n* = 1), Remethylierungsstörung (*n* = 1) und GLUT1-Mangel (*n* = 1).

Die genetische Diagnostik erbrachte in 41,4 % (12/29) pathologische Befunde; darunter bei 5/12 (41,7 %) größere Mutationen, bei 4/12 (33,3 %) Mutationen in spezifischen Genen (*ATRX*-Gen, *YWHAE*-Gen, *MTFTMT*-Gen, *TSC2*-Gen) und bei 3/12 (25 %) ein genetisches Syndrom (Wardenburg-Syndrom Typ 1, Coffin-Siris-Syndrom, Fragiles-X-Syndrom).

Der Vergleich von Kindern mit motorischer Entwicklungsverzögerung (*n* = 30) mit Kindern ohne motorische Entwicklungsverzögerung (*n* = 44) zeigte signifikant häufiger pathologische Befunde für die folgenden Parameter (Tab. 1 und 2): Komorbiditäten (83,3 % vs. 56,8 %; *p* = 0,008), internistische Untersuchung (53,3 % vs. 29,5 %; *p* = 0,047) und Augenuntersuchung (36,7 % vs. 11,4 %; *p* = 0,049). Die restlichen Parameter zeigten keine signifikanten Unterschiede zwischen beiden Gruppen.

## Diskussion

Das Hauptziel der hier vorliegenden retrospektiven Studie war die Darstellung des diagnostischen Prozesses und der Ergebnisse der neuropädiatrischen Untersuchungen im Rahmen einer Autismusabklärung.

In unserem Patientenkollektiv, welches 82 Patienten umfasst, zeigte sich ein Geschlechterverhältnis von männlich zu weiblich von 3,5:1, was dem in der Literatur beschriebenen Verhältnis von 2–3:1 entspricht [15, 22]. Das Durchschnittsalter bei Vorstellung in unserer neuropädiatrischen Ambulanz betrug 5,9 Jahre, wobei die meistvertretene Altersgruppe zwischen 3 und 5 Jahren lag. Wann genau die Autismusdiagnose gestellt wurde, ließ sich jedoch in unserem Kollektiv nicht durchgängig ermitteln. Rund die Hälfte der ASS-Diagnosen wurde nicht näher klassifiziert, was die Notwendigkeit einer Anpassung der Diagnosekriterien und Einteilung unterstreicht, wie sie in der zukünftig geltenden ICD-11 auch vorgesehen ist.

In unserer Studienpopulation war weit mehr als die Hälfte (64,6 %) von somatischen und psychischen Komorbiditäten betroffen. Diese Beobachtung deckt sich beispielsweise auch mit den Ergebnissen von Rosen et al., die in 63–78 % mindestens eine psychische Komorbidität fanden und in 10–77 % mindestens eine somatische Begleiterkrankung [29]. Da viele Begleiterkrankungen bereits durch eine detaillierte Anamnese und gründliche klinisch-neurologische Untersuchung erkannt oder differenzialdiagnostisch ausgeschlossen werden können bzw. sich hieraus Indikationen für weiterführende Untersuchungen ergeben, sollte dies bei jedem Kind mit Verdacht auf ASS sorgfältig durchgeführt werden.

In unserer Studie wurde bei 33,8 % (25/74) der Kinder ein auffälliges EEG festgestellt. Bei 19,5 % (16/82) wurde anhand des EEGs und/oder der Anamnese eine Epilepsie erfasst; 2 Kinder zeigten ein unauffälliges EEG trotz bekannter Epilepsie. Die genaue Prävalenz von Epilepsie bei Autismus ist nicht bekannt; in Studien variieren die Zahlen von 5–46 % [34, 37].

Bei 14,9 % derer, die ein EEG erhielten, zeigte sich in unserer Kohorte ein auffälliger Befund ohne klinisches Korrelat oder diagnostizierte Epilepsie. Chez et al. fanden bei 889 Patienten mit ASS in 61 % Abweichungen im EEG ohne bekannte Krampfanfälle [4]. Andere Studien hingegen berichten von lediglich 22 % bzw. 32 % [1]. Ein möglicher Grund für die hohe Prävalenz in der Studie von Chez et al. [4] könnte sein, dass sie die Ergebnisse von 24-h-EEGs erfassten. Bei Patienten mit normalen Routine-EEGs könnte ca. die Hälfte an Auffälligkeiten übersehen werden [4].

Die Bedeutung von Auffälligkeiten im EEG und damit auch die Notwendigkeit der Durchführung eines EEGs muss auch im Hinblick auf epileptische Enzephalopathien bewertet werden. Dieser Begriff beschreibt, dass es aufgrund der epileptischen Aktivität zu kognitiven Störungen und Verhaltensauffälligkeiten, einschließlich ASS, kommt, die über die zugrunde liegende, oft genetisch bedingte Pathologie hinausgehen [27]. Unter der Annahme, dass die epileptischen Aktivitäten im Zusammenhang mit den Verhaltensauffälligkeiten, Sprachdefiziten und eingeschränkter kognitiver Leistung stehen, ist es möglich, dass sich eine antikonvulsive Therapie auch ohne das Auftreten von Krampfanfällen durch eine Normalisierung des EEGs positiv auf die Symptomatik bei ASS auswirken kann [27, 34]. Diese Erkenntnisse stellen die Empfehlungen bezüglich der Durchführung eines EEGs in der aktuell gültigen Leitlinie zur Diagnostik bei ASS infrage. Diese empfiehlt, nur bei klinischer Indikation, aufgrund der Anamnese und internistisch/neurologischer Untersuchung ein EEG durchzuführen [7].

Zum jetzigen Zeitpunkt überwiegt für uns der Nutzen, das EEG als kostengünstige und wenig belastende Untersuchung in die Routinediagnostik bei ASS aufzunehmen; allerdings ist teilweise auch die Gabe von Beruhigungsmitteln notwendig [16, 20]. Aufgrund der bekannten zweigipfligen Erstmanifestation der Epilepsie empfehlen wir die Durchführung bei jedem Kind mit Verdacht auf ASS vor dem 5. Lebensjahr und im Verlauf nach dem 10. Lebensjahr.

In unserer Studie zeigte sich bei 22/49 (44,9 %) Kindern mindestens ein auffälliges Ergebnis in der cMRT-Untersuchung. Bei 14/22 (63,8 %) konnten die Befunde als eindeutige Pathologien im Sinne von Fehlbildungen oder Läsionen klassifiziert werden. Ähnliche Arbeiten erfassten mit 12 % bzw. 17 % deutlich weniger strukturelle Auffälligkeiten des Gehirns [5, 39]. Es ist jedoch anzumerken, dass in unserem Kollektiv bei 60 % der Patienten eine cMRT durchgeführt wurde, wohingegen bei Voigt et al. und Chudley et al. lediglich bei 20 % bzw. 13 % eine cMRT oder eine Computertomographie erfolgte [5, 39].

Darüber hinaus fand sich bei 55,5 % der Kinder mit Mikro- oder Makrozephalie, bei 53,8 % der Kinder mit Epilepsie und 50,0 % der Patienten mit auffälligem EEG eine auffällige cMRT. Auch Cooper et al. registrierten in ihrer Studie zur Bedeutung des cMRTs bei ASS mehr Pathologien bei klinischen Auffälligkeiten. So kamen sie zu dem Ergebnis, dass die Prävalenz auf bis zu 26,2 % steigt, wenn klinische Auffälligkeiten wie eine auffällige neurologische Untersuchung oder Epilepsie vorliegen. Hingegen fanden sie beim alleinigen Vorliegen einer ASS lediglich eine Prävalenz von 6,5 %. Diese Erkenntnisse unterstützen die Empfehlung der aktuellen Leitlinie, nicht standardmäßig bei jedem Kind eine cMRT durchzuführen [6, 7]. Ein Grund für die höhere Prävalenz in unserer Studie könnte sein, dass in unserem Kollektiv auch Auffälligkeiten erfasst wurden, die nicht eindeutigen Pathologien wie Fehlbildungen, Läsionen oder Tumoren zugeordnet werden konnten.

In unserer Studie erhielt mehr als die Hälfte (53,7 %) der Kinder ein Stoffwechselscreening. Bei 11,4 % (5/44) derer, die ein Stoffwechselscreening erhalten hatten, konnte letztlich eine Stoffwechselerkrankung bzw. der Verdacht darauf diagnostiziert werden. Damit liegt die Prävalenz für Stoffwechselerkrankungen bei Kindern mit ASS in unserer Studie deutlich höher als in der Literatur mit 0–5 % angenommen [24, 33]. Ursache für die deutliche Differenz könnte in unseren Einschlusskriterien liegen, aufgrund derer kein Ausschluss bei Vorliegen eines Syndroms erfolgte. Dementsprechend ist anzunehmen, dass die höhere Prävalenz in unserer Studie dem Einschluss von Patienten mit syndromalem Autismus geschuldet ist.

Unter Berücksichtigung der Tatsache, dass sich einige Stoffwechselerkrankungen therapieren lassen und somit die Prognose positiv beeinflusst werden kann, sollte aus unserer Sicht eine Stoffwechseldiagnostik durchgeführt werden, wenn durch vorhergehende gründliche klinische Untersuchung und Anamnese Hinweise auf einen syndromalen Autismus erkannt werden. „Red Flags“, die zu Laboruntersuchungen führen sollten, sind: Lethargie, zyklisches Erbrechen, Krampfanfälle, Dysmorphiezeichen, mentale Retardierung, auffälliges Neugeborenenscreening, Geburt in einem Land, welches kein Neugeborenenscreening durchführt, Elektrolytstörungen, gastrointestinale Störungen, Hypotonie oder Sprachregression [11, 24, 32]. Zudem treten Stoffwechselerkrankungen durch die oftmals autosomal-rezessive Vererbung vermehrt in Ländern mit hohem Anteil an blutsverwandten Eltern auf, weshalb auch dies einen Grund für eine metabolische Diagnostik darstellen sollte [11].

Die Empfehlungen einer genetischen Untersuchung im Rahmen der Abklärung einer ASS sind nicht einheitlich. Einige Autoren empfehlen, eine genetische Diagnostik bei jedem Kind durchzuführen, und beziehen sich auf die Richtlinien des American College of Medical Genetics (ACMG) von 2013. In der aktuellen S3-Leitlinie der AWMF wird in der konsensbasierten Empfehlung eine humangenetische Untersuchung nur bei klinischer Indikation empfohlen [7].

Obwohl in unserer Studie bei 60 % eine genetische Abklärung empfohlen wurde, lagen bei lediglich 35 % (29/82) Ergebnisse diesbezüglich vor. Auffällige Befunde zeigten sich bei 41 % (12/29). Dies liegt damit deutlich über den Ergebnissen einer ähnlich aufgebauten Studie, in der nur bei 6 % (6/97) eine auffällige Chromosomenanalyse sowie bei 2 % (2/104) Veränderungen in der DNA im Sinne eines Fragilen-X-Syndroms gefunden wurden [39]. In unserer genetischen Abklärung wurden allerdings je nach Einschätzung des Humangenetikers neben der Karyotypisierung und der Array-CGH auch einzelne Gensequenzierungen sowie PANEL-Sequenzierungen durchgeführt. Dies könnte ein Grund für den höheren diagnostischen Ertrag sein. So ist anzunehmen, dass je nach individueller Einschätzung der behandelnden Ärzte sowie der Verfügbarkeit an Laboruntersuchungen, der Umfang und damit auch der diagnostische Ertrag der genetischen Diagnostik variiert [7].

Auch das Wiederholungsrisiko für die Familien spielt bei der genetischen Diagnostik eine große Rolle. In unserer Studie lag anamnestisch bei 13,4 % eine positive Familienanamnese für Autismus vor. Bei selektiver Betrachtung einer positiven Familienanamnese für Autismus bei Geschwistern, lag die Prävalenz in unserer Studie bei 6 %. Dies deckt sich mit Daten aus der Literatur, die das Wiederholungsrisiko bei Geschwistern mit 3,9–6,3 % beziffern [3, 14]. Ob hier Veränderungen in gleichen Genen ursächlich sind, wurde in unserer Studie nicht erfasst und müsste Gegenstand weiterer Untersuchungen sein. In Zukunft dürfte wohl auch der Exom-Analyse – sowohl für wissenschaftliche als auch für klinische Fragestellungen – eine zunehmend größere Bedeutung im Rahmen der ASS-Diagnostik zukommen [41, 42].

Bei Betrachtung der Kinder mit und ohne motorische Entwicklungsverzögerung fällt v. a. auf, dass eine motorische Entwicklungsverzögerung häufiger mit Komorbiditäten, EEG-Auffälligkeiten und Auffälligkeiten in der Stoffwechseluntersuchung und der genetischen Diagnostik assoziiert war, auch wenn nicht in allen Punkten signifikant. Wir plädieren daher dafür, dass bei jedem Kind im Rahmen der ASS-Diagnostik, ein EEG durchgeführt werden sollte. Da bei der hohen Prävalenz von bis zu 46 % für eine Epilepsie im Rahmen der ASS [34, 37] und der potenziell guten Behandelbarkeit bis hin zur Anfallsfreiheit auch ein Gewinn an Lebensqualität für Patienten und deren Familien erreicht werden kann, erscheint uns dieses Vorgehen gerechtfertigt. Darüber hinaus rechtfertigt häufiges Auftreten von epilepsietypischen Veränderungen ohne klinisches Korrelat im Hinblick auf epileptische Enzephalopathien die standardmäßige Durchführung eines EEGs [4, 27].

Weiterführende Abklärungen mittels cMRT, Stoffwechseldiagnostik und/oder genetischer Diagnostik sollten hingegen nicht standardmäßig bei jedem Patienten mit Verdacht auf ASS empfohlen werden. Hier richtet sich das weitere Prozedere insbesondere nach den Befunden aus der internistisch-neurologischen Untersuchung, der Anamnese, EEG-Pathologien und dem Vorliegen von „Red Flags“.

Die Durchführung eines Hörscreenings und eines Sehtests ist nicht Teil unseres diagnostischen Prozesses, sondern wird in der Regel vor Überweisung in unsere neuropädiatrische Ambulanz von externen Kollegen veranlasst. Wir befürworten aber den Ausschluss von Hör- und Sehstörungen als mögliche Ursache für die autistischen Symptome.

Eine wesentliche Limitation unserer Studie stellt das retrospektive Studiendesign dar mit den entsprechenden Auswirkungen auf die Datenerhebung, -auswertung und -interpretation. Des Weiteren stellen die kleine Gruppengröße und die monozentrische Erhebung der Daten eine weitere Einschränkung dar, die die statistische Aussagekraft beschränken.

Aufgrund der erhobenen Daten sollte aus unserer Sicht bei jedem Patienten mit Verdacht auf ASS eine Basisdiagnostik aus detaillierter Anamnese, einer internistisch-neurologischen Untersuchung sowie einer EEG erfolgen. Die Durchführung weiterer Untersuchungen wie einer cMRT, einer stoffwechsel- sowie einer genetischen Diagnostik wird hingegen nicht standardmäßig empfohlen. Dagegen sollte deren Indikation individuell anhand der erhobenen Befunde aus der Basisdiagnostik gestellt werden.

## Figures and Tables

**Abb. 1:**
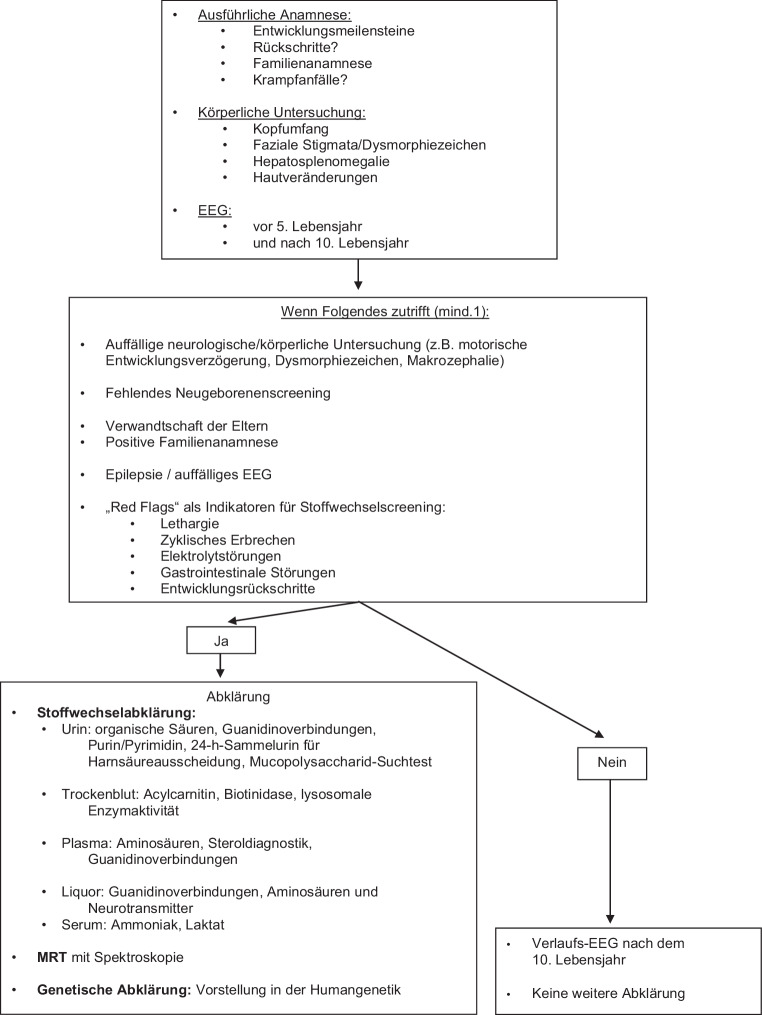
Handlungsempfehlung (SOP) für die diagnostische Vorgehensweise bei Vorstellung mit Verdacht auf Autismus in der neuropädiatrischen Ambulanz

## Anhang

​

**Tab. 1:** Patientencharakteristika, dargestellt in absoluten und relativen Häufigkeiten

	Motorische Entwicklungsverzögerung	Keine motorische Entwicklungsverzögerung	Gesamt	*p*-Wert
*Autismusform*	–	–	–	0,52
Nicht weiter klassifizierte ASS	17/30 (56,7 %)	19/44 (43,2 %)	36/74 (48,6 %)	–
Frühkindlicher Autismus	6/30 (20,0 %)	17/44 (38,6 %)	23/74 (31,1 %)	
Atypischer Autismus	5/30 (16,7 %)	6/44 (13,6 %)	11/74 (14,9 %)	
Asperger-Syndrom	2/30 (6,7 %)	2/44 (4,5 %)	4/74 (5,4 %)	
*Auffällige Familienanamnese*	12/30 (40 %)	18/44 (40,9 %)	–	0,88
Autismus	–	–	11/30 (36,7 %)	–
Lernschwäche			9/30 (30,0 %)	
Epilepsie			5/30 (16,7 %)	
Mentale Retardierung			5/30 (16,7 %)	
Genetische Erkrankung			4/30 (13,3 %)	
*Auffälligkeiten in der Schwangerschaft*	10/30 (33,3 %)	11/44 (25,0 %)	–	0,39
Geburtskomplikationen	–	–	7/21 (33,3 %)	–
Frühgeburtlichkeit			6/21 (28,6 %)	
Nikotin/Alkohol			4/21 (19,0 %)	
Komorbidität der Mutter			4/21 (19,0 %)	
Psychische Belastung			3/21 (14,3 %)	
Intrauterine Auffälligkeiten			3/21 (14,3 %)	
Andere			3/21 (14,3 %)	
*Entwicklungsstand*	–	–	–	–
Sprachliche Entwicklungsverzögerung	26/30 (86,7 %)	40/44 (90,9 %)	70/82 (85,4 %)	0,71
Motorische Entwicklungsverzögerung	–	–	30/82 (36,5 %)	–
Intelligenzminderung	17/44 (56,7 %)	14/44 (31,8 %)	32/82 (39,0 %)	0,32
*Somatische Komorbiditäten*	25/33 (83,3 %)	25/44 (56,8 %)	–	0,008
Epilepsie	–	–	16/50 (19,5 %)	–
Syndrome/Fehlbildungen			12/50 (14,6 %)	
Fußfehlhaltung			11/50 (13,4 %)	
Herzfehler			8/50 (9,8 %)	
Andere			6/50 (7,3 %)	
Krampfanfälle			5/50 (6,0 %)	
Hormonstörung			4/50 (4,9 %)	
Stoffwechselstörung			4/50 (4,9 %)	
Zustand nach Infektion			3/50 (3,7 %)	
Ernährungsintoleranz			2/50 (2,4 %)	
Kopfschmerzen			2/50 (2,4 %)	
*Psychomotorische Komorbiditäten*	–	–	–	–
Aggressivität/geringe Frustrationsbereitschaft	–	–	16/82 (19,5 %)	–
Andere			15/82 (18,3 %)	
Schlafstörung			13/82 (15,9 %)	
Ausscheidungsstörung			10/82 (12,2 %)	
Hyperaktivität/verminderte Aufmerksamkeit			8/82 (9,8 %)	
Essstörung			6/82 (7,3 %)	
Ängstlichkeit			5/82 (6,1 %)	

Entwicklungsstand: Unter „Komorbidität der Mutter“ wurden Schwangerschaftsdiabetes, Epilepsie und ein Alter über 35 Jahren zusammengefasst. „Andere“ beinhaltet Blutungen während der Schwangerschaft und Eisenmangel der Mutter

Somatische Komorbiditäten: „Zustand nach Infektion“ beinhaltet Beginn der autistischen Symptomatik nach Entzündung (Enzephalitis oder Mittelohrentzündung)

Psychische Komorbiditäten: Unter der Kategorie „Andere“ sind Ticstörungen, psychomotorische Unruhe und Lernbehinderungen zusammengefasst

Vergleich kategorialer Zielgrößen mittels Chi^2^-Test; Signifikanzniveau wurde auf *p* < 0,05 festgelegt

**Tab. 2:** Klinische und apparative Diagnostik, dargestellt als absolute und relative Häufigkeiten

	Motorische Entwicklungsverzögerung	Keine motorische Entwicklungsverzögerung	Gesamt	*p*-Wert
**Internistische Untersuchung**	–	–	70/82 (95,1 %)	–
*Pathologischer Untersuchungsbefund*	16/30 (53,3 %)	13/44 (29,5 %)	30/78 (38,5 %)	0,47
Dysmorphiezeichen	–	–	13/30 (43,3 %)	–
Makrozephalie/Adipositas			3/30 (10,0 %)	
Hautstigmata			8/30 (26,7 %)	
**Neuropädiatrische Untersuchung**	–	–	78/82 (95,1 %)	–
*Pathologischer Untersuchungsbefund*	28/30 (93,3 %)	40/44 (90,9 %)	72/78 (92,3 %)	0,15
Sprache	–	–	67/78 (85,9 %)	–
Soziale Interaktion			46/78 (59,0 %)	
Stereotypen			33/78 (42,3 %)	
Grobmotorik			24/78 (30,8 %)	
Feinmotorik			19/78 (24,4 %)	
Koordination			17/78 (21,8 %)	
Muskelhypotonie			13/78 (16,7 %)	
Muskeleigenreflexe			6/78 (7,7 %)	
**Augenärztliche Untersuchung**	–	–	32/82 (39,0 %)	–
*Pathologischer Untersuchungsbefund*	11/30 (36,7 %)	5/44 (11,4 %)	17/32 (53,1 %)	0,049
Amblyopie	–	–	13/17 (76,5 %)	–
Strabismus			7/17 (41,2 %)	
Augenhintergrundveränderung			4/17 (23,5 %)	
Nystagmus			2/17 (11,8 %)	
**Elektroenzephalographie**	–	–	74/82 (90,2 %)	–
*Pathologische Untersuchungsbefunde*	12/30 (40,0 %)	12/44 (27,3 %)	25/74 (33,8 %)	0,28
Fokale ETPs	–	–	20/25 (80,0 %)	–
Generalisierte ETPs			2/25 (8,0 %)	
Herdbefund			2/25 (8,0 %)	
Fokale ETPs mit Generalisierungstendenz			1/25 (4,0 %)	
*Epilepsie im EEG*	8/30 (26,7 %)	7/44 (15,9 %)	14/16 (87,5 %)	0,26
*Epilepsieformen*	–	–	–	–
Rolando-Epilepsie	–	–	3/16 (18,8 %)	–
Absence-Epilepsie			2/16 (12,5 %)	
Myoklonische Epilepsie			1/16 (6,3 %)	
Benigne Partialepilepsie			1/16 (6,3 %)	
Frontallappenepilepsie			1/16 (6,3 %)	
Temporallappenepilepsie			1/16 (6,3 %)	
WEST-Syndrom			1/16 (6,3 %)	
Nicht näher bezeichnet			6/16 (37,5 %)	
**Kraniale Magnetresonanztomographie**	–	–	49/82 (59,8 %)	–
*Pathologische Befunde*	9/30 (30,0 %)	13/44 (29,5 %)	30/82 (36,6 %)	0,74
Sonstige	–	–	14/30 (46,7 %)	–
Läsionen			8/30 (26,7 %)	
Fehlbildungen			7/30 (23,3 %)	
Tumoren			1/30 (3,3 %)	
**Stoffwechseldiagnostik**	–	–	44/82 (53,7 %)	–
*Pathologische Befunde*	7/30 (23,3 %)	5/44 (11,4 %)	13/44 (29,5 %)	0,38
Laktat	–	–	7	–
Aminosäuren im Plasma			4	
Ammoniak			3	
Organische Säuren im Urin			3	
Aminosäuren im Liquor			2	
Guanidinoverbindungen im Plasma			1	
Carnitin im Plasma			1	
Acylcarnitinprofil im Trockenblut			0	
Gesamtglykosaminoglykane im Urin			0	
Guanidinoverbindungen im Urin			0	
**Genetische Testung**	–	–	29/82 (35,4 %)	–
*Pathologische Befunde*	7/30 (23,3 %)	4/44 (9,1 %)	12/29 (41,4 %)	0,18
Größere Mutationen	–	–	5/12 (41,7 %)	–
Mutationen in spezifischen Genen			4/12 (33,3 %)	
Genetisches Syndrom			3/12 (25,0 %)	

Vergleich kategorialer Zielgrößen mittels Chi^2^-Test

Signifikanzniveau wurde auf *p* < 0,05 festgelegt
